# Supplementary material for: Functional control of oscillator networks
Source: Nat Commun. 2022 Aug 11;13:4721. doi: 10.1038/s41467-022-31733-2 (PMC9372149; doi:10.1038/s41467-022-31733-2)
Supplement: Supplementary file 1 — Supplementary Information [file 41467_2022_31733_MOESM1_ESM.pdf]

Supplementary Information to

Functional Control of Oscillator Networks

Tommaso Menara
Department of Mechanical and Aerospace Engineering, University of California, San Diego, La Jolla, CA 92093, USA

Giacomo Baggio
Department of Information Engineering, University of Padova, Padova, 35131, Italy

Danielle S. Bassett
Department of Physics & Astronomy, Department of Bioengineering, Department of Electrical & Systems Engineering, Department of Neurology, Department of Psychiatry, University of Pennsylvania, Philadelphia, PA 19104, USA
The Santa Fe Institute, Santa Fe, NM 87506, USA

Fabio Pasqualetti
Department of Mechanical Engineering, University of California, Riverside, Riverside, CA 92521, USA
fabiopas@engr.ucr.edu

Contents

1 Supplementary Text **3**

1.1 Comparison between our local order parameter and the Pearson correlation coefficient 3

1.2 Stability results for functional patterns with angle differences larger than $\frac{\pi}{2}$ in the case of line and cycle with positive weights 3

1.3 Functional patterns possess at most almost-global stability in positive cluster-synchronized networks 4

1.4 Allocation of multiple phase-locked equilibria by tailoring of the network structural parameters . 6

1.5 A heuristic method to promote stability of functional patterns in positive networks 6

1.6 Extension of the proposed optimization methods to directed networks 8

1.7 Coupled Kuramoto oscillators to approximate fMRI data 10

1.8 Procedure to extract phase-locked trajectories from fMRI data 10

1.9 Power network modeling and assumptions 11

1.10 Application to additional power network models 11

2 Supplementary Figures **16**

1 Supplementary Text

1.1 Comparison between our local order parameter and the Pearson correlation coefficient

In this work, we provide methods and principles to guarantee the emergence of phase-locked trajectories that describe functional patterns. The latter are defined utilizing $\rho_{ij} =< \cos(\theta_j - \theta_i) >_t$, instead of the classical Pearson correlation coefficient. The same local order parameter is also utilized in [7] to quantify the similarity between BOLD signals in functional MRI recordings, and in [1], to quantify the synchrony in hierarchical networks.

To compare our metric and the Pearson correlation coefficient, recall the definition of the latter, which is a measure of linear correlation between two series of data. For vectors of samples $y \in \mathbb{R}^T$ and $z \in \mathbb{R}^T$:

$$r_{ij} = \frac{\sum_{i=1}^{N}(y_i - y_{\text{mean}})(z_i - z_{\text{mean}})}{\sqrt{\sum_{i=1}^{N}(y_i - y_{\text{mean}})^2}\sqrt{\sum_{i=1}^{N}(z_i - z_{\text{mean}})^2}},$$

where y_{mean} and z_{mean} denote the sample means of vectors y and z, respectively. Notice that the length T of the vectors y and z depends on the sampling time and on the window length. We argue that our local order parameter (equation (2) in the main text) is simply more convenient when dealing with periodic phase signals. While we could use the Pearson correlation coefficient to define functional pattern instead of our cosine-based local order parameter, the former does not perform well on periodic signals that evolve on the unit circle, and is heavily dependent on the time window employed to collect the samples. Thus, specific adjustments are needed to define correlation patterns through Pearson correlation coefficient for the class of time series (phase trajectories) studied in this work.

As an example, consider two identical sinusoidal signals (i.e., phase-locked) with natural frequency $\omega = 2\pi$ but shifted initial conditions: $\theta_1(0) = 0$, $\theta_2(0) = \varphi$, with $\varphi \in (0,\ \pi]$. Fig. 1 illustrates the differences between the Pearson correlation coefficient r_{12} and our local order parameter ρ_{12} computed over a time window of varying length and for different values of the initial phase shift φ. In all panels, the values of r_{12} vary at each point, emphasizing the dependence of the Pearson correlation coefficient from the length of the time window. Conversely, in all panels, the value of ρ_{12} remains unaltered by the choice of time window length.

In conclusion, we choose to define functional patterns through $\rho_{ij} =< \cos(\theta_j - \theta_i) >_t$ because it is convenient and suitable for this class of phase trajectories that naturally emerge in oscillator systems.

1.2 Stability results for functional patterns with angle differences larger than $\frac{\pi}{2}$ in the case of line and cycle with positive weights

Consider a line network of n (ordered) oscillators with positive-only weights that possesses an equilibrium for the phase difference dynamics satisfying $|x_{i,i+1}| > \frac{\pi}{2}$ for some $i \in \{1, \ldots, n-1\}$. It is straightforward to deduce from the results on structural balance [32] that the considered equilibrium is unstable. This implies that the functional pattern associated with that equilibrium is unstable.

Intuitively, the simplest topology that lends itself to a characterization of stable phase configurations including $|x_{i,j}| > \frac{\pi}{2}$ and allowing only positive weights is the cycle (i.e., a line network where the first and last oscillators are connected). Consider a cycle network of $n > 4$ oscillators with positive weights, and denote with $\boldsymbol{x}_{\text{cycle}}$ a vector of the n phase differences between connected oscillators. We find that, after a suitable relabeling of the oscillators for which x_{12} satisfies $|x_{12}| > \frac{\pi}{2}$:

Theorem 2 ***(Stability of phase differences equilibria with $|x_{ij}| > \frac{\pi}{2}$ in cycle networks)*** *The equilibrium $\boldsymbol{x}_{\text{cycle}} = [x_{12}\ x_{23}\ \ldots\ x_{n-1,n}]^{\mathsf{T}} = [\gamma\ \varphi_1\ \ldots\ \varphi_{n-1}]^{\mathsf{T}}$ with $|\gamma| > \frac{\pi}{2}$, $|\varphi_i| < \frac{\pi}{2}$, and $\varphi_i = \varphi_{n-i}$ for all $i = 1, \ldots, n-1$, is stable if and only if*

(i) $A_{i,i+1} = A_{12}\frac{\sin(\gamma)}{\sin(\varphi_{i-1})}$
for $i = 2, \ldots, n$, *with* $n - i + 3 \triangleq 1$ *if* $i = 2$, *and* $n + 1 \triangleq 1$;

(ii) $|\cot\gamma| \leq (\tan(\varphi_1) + \cdots + \tan(\varphi_{n-1}))^{-1}$.

Moreover, if $\varphi_1 = \cdots = \varphi_{n-1}$ *and* $n \to \infty$, *the largest possible value for* γ *such that* $\boldsymbol{x}_{\text{cycle}}$ *is stable tends to the value* $\gamma \approx 1.789776$, *which is the solution to* $\gamma - \tan(\gamma) = 2\pi$.

Proof of Theorem 2: To assess the stability of the equilibrium $\bar{\boldsymbol{x}}_{\text{cycle}} = [x_{12}\ x_{23}\ \ldots\ x_{n-1,n}]^{\mathsf{T}} = [\gamma\ \varphi_1\ \ldots\ \varphi_{n-1}]^{\mathsf{T}}$ with $|\gamma| > \frac{\pi}{2}$, $|\varphi_i| < \frac{\pi}{2}$, and $\varphi_i = \varphi_{n-i}$ for all $i = 1, \ldots, n-1$, we analyze the spectrum of the Jacobian $J(\boldsymbol{x}_{\text{cycle}}) = -\mathcal{L}(\boldsymbol{x}_{\text{cycle}})$. From Ref. [32, Corollary IV.7], we have that a necessary and sufficient condition for the Laplacian matrix $\mathcal{L}(\boldsymbol{x}_{\text{cycle}})$ of the cosine-scaled network to be positive semidefinite is

$$|A_{12}\cos(\gamma)| \leq \mathcal{R}_{12}^{-1}, \tag{1}$$

with $\mathcal{R}_{12}$ being the effective resistance of the graph in which the edge $(1, 2)$ has been removed. That is,

$$\mathcal{R}_{12} = \frac{1}{A_{23}\cos(\varphi_1)} + \cdots + \frac{1}{A_{n-1,n}\cos(\varphi_{n-1})}. \tag{2}$$

Since the adjacency matrix satisfies $A = A^{\mathsf{T}}$ and $\boldsymbol{x}_{\text{cycle}}$ is an equilibrium for the difference dynamics of the cycle network, the network weights must be identical pairwise:

$$A_{i,i+1} = A_{n-i+2,n-i+3} = A_{12}\frac{\sin(\gamma)}{\sin(\varphi_{i-1})}, \tag{3}$$

for $i = 2, \ldots, n$ with the convention $n - i + 3 \triangleq 1$ if $i = 2$. Thus, the angles being identical pairwise implies that the network weights must also be identical pairwise, which yields condition (i) of Theorem 2. Moreover, plugging the network weights from equation (3) into equation (2) yields

$$\mathcal{R}_{12} = \frac{\tan(\varphi_1)}{A_{12}\sin(\gamma)} + \cdots + \frac{\tan(\varphi_{n-1})}{A_{12}\sin(\gamma)}, \tag{4}$$

which makes the condition in Eq. (1) become, after algebraic calculations, condition (ii) of Theorem 2:

$$|\cot\gamma| \leq (\tan(\varphi_1) + \cdots + \tan(\varphi_{n-1}))^{-1}. \tag{5}$$

Thus, given that the Laplacian $\mathcal{L}(\boldsymbol{x}_{\text{cycle}})$ is positive definite, the Jacobian $J(\boldsymbol{x}_{\text{cycle}})$ is stable, and its only zero eigenvalue is due to rotational symmetry of the right-hand side of the Kuramoto dynamics (equation (2) of the main manuscript).

For $\varphi_1 = \cdots = \varphi_{n-1}$, we have that

$$\varphi_i = \frac{2\pi - \gamma}{n-1}.$$

Hence, the right-hand side of equation (5) becomes $\frac{\cot(\varphi)}{n-1}$, and $\lim_{n\to\infty}\frac{\cot(\varphi)}{n-1} = \frac{1}{2\pi-\gamma}$. Since $|\gamma| > \frac{\pi}{2}$, plugging the limit value for $\frac{1}{2\pi-\gamma}$ into equation (5) and solving for the equality yields $\gamma - \tan(\gamma) = 2\pi$, whose unique solution is $\gamma \approx 1.789776$. This concludes the proof. ■

1.3 Functional patterns possess at most almost-global stability in positive cluster-synchronized networks

In general, the analysis and estimation of the basin of attraction of nonlinear systems remains an outstanding problem, and even the most recent results rely on numerical approaches or heavy modeling assumptions [5]. To

the best of our knowledge, Ref. [33] provides the most up-to-date study of the basins of attraction of synchronized Kuramoto oscillators. However, the authors in Ref. [33] only derive estimates of the basin of attraction for the fully synchronized case in networks of identical oscillators. Below, we show that functional patterns associated with phase-locked trajectories in cluster-synchronized positive networks are at most almost-globally stable. Our results extend previous work on identical oscillators to the case of oscillators with heterogeneous natural frequencies.

Existing literature shows that the number of equilibria for the phase differences of heterogeneous oscillators evolving on random graphs increases significantly with the cardinality of the network [17]. Therefore, we begin by restricting our analysis to the case of identical oscillators. In general, for any connected network of identical oscillators with cooperative connections $A_{ij} \geq 0$, there is no unique pattern. In fact, the largest basin of attraction is achieved by $\mathbb{S}^1$-*synchronizing* graphs (e.g., complete graphs, acyclic graphs, and sufficiently dense graphs [10,29]), which feature almost-global stability of the fully synchronized functional pattern. In this class of networks, the only stable equilibrium $\boldsymbol{x}$ satisfies $x_{ij} = 0$ for all i, j, and there exists a finite number of unstable equilibria. For instance, consider two connected identical oscillators: $\omega_1 = \omega_2$. The only stable equilibrium for the phase differences dynamics is $x_{12} = \theta_2 - \theta_1 = 0$. Yet, there also exists an unstable equilibrium $x_{12} = \pi$. Note that, in general, almost-global stability cannot be guaranteed for arbitrary classes of sparse networks of identical oscillators, as other synchronization manifolds besides the fully synchronized one can be stable – for example, splay states emerge in Cayley graphs [16]. This latter class of graphs admits two stable equilibria (full synchronization and splay states), which implies the coexistence of two stable patterns.

We can generalize the above observations to networks of heterogeneous oscillators. To do so, we leverage cluster synchronization – a phenomenon where distinct groups of synchronized oscillators coexist in a network [19] – of $\mathbb{S}^1$-*synchronizing* clusters with possibly different natural frequencies. We consider cluster synchronization in networks with a partition of the oscillators $\mathcal{C} = \{\mathcal{C}_1, \ldots, \mathcal{C}_m\}$, with $\mathcal{C}_k \subseteq \mathcal{O}$ being a subset of the network oscillators constituting an $\mathbb{S}^1$-*synchronizing* graph, $k \in \{1, \ldots, m\}$, where $\bigcup_{k=1}^m \mathcal{C}_k = \mathcal{O}$ and $\mathcal{C}_k \cap \mathcal{C}_\ell = \emptyset$ if $k \neq \ell$. Further, $\omega_i = \omega_j$ for every $i, j \in \mathcal{C}_k$, $k \in \{1, \ldots, m\}$.[1] Whenever cluster synchronization emerges, the diagonal blocks (of sizes $|\mathcal{C}_k| \times |\mathcal{C}_k|$, $k = 1, \ldots, m$) of the functional pattern associated with partition $\mathcal{C}$ satisfy $\rho_{ij} = 1$ (see Supplementary Fig. 6).[2] Thus, for a given number of clusters $m \geq 1$, each of the $\binom{n}{m}$ possible ways to partition the network yields a functional pattern with diagonal blocks corresponding to synchronized clusters.

By extending the above observations on the stability of $\mathbb{S}^1$-*synchronizing* graphs, we find that, in networks where at least one cluster satisfies $|\mathcal{C}_k| \geq 2$, at most almost-global stability of cluster-synchronized functional patterns can be achieved. In fact, for any choice of intra-cluster phase differences from the finite set of unstable equilibria in $\mathbb{S}^1$-*synchronizing* graphs (i.e., $x_{ij} = \pi$ for at least one intra-cluster phase difference), there exists a value for the inter-cluster phase differences for which the network admits phase-locked trajectories where intra-cluster phase differences satisfy $x_{ij} = 0$ or $x_{ij} = \pi$.

As an example, consider a 4-oscillator network partitioned as $\mathcal{C} = \{\mathcal{C}_1, \mathcal{C}_2\}$, where $\mathcal{C}_1 = \{1, 2\}$ and $\mathcal{C}_2 = \{3, 4\}$. The network parameters read

$$A = \begin{bmatrix} 0 & 5 & 2 & 0 \\ 5 & 0 & 0 & 2 \\ 2 & 0 & 0 & 6 \\ 0 & 2 & 6 & 0 \end{bmatrix} \quad \text{and} \quad \boldsymbol{\omega} = \begin{bmatrix} 1 \\ 1 \\ 2 \\ 2 \end{bmatrix}.$$

Note that the two clusters are $\mathbb{S}^1$-*synchronizing* graphs. It can be shown that there exists an unstable equilibrium at $\boldsymbol{x}_{\text{desired}} = [x_{12}\ x_{23}\ x_{34}]^\mathsf{T} = [\pi\ 0.25268\ \pi]^\mathsf{T}$. Thus, whenever the pattern R associated to the partition $\mathcal{C}$ is stable, it is at most almost-globally stable.

[1] This is a necessary and sufficient condition for cluster synchronization [19].

[2] Without loss of generality, we assume that the network oscillators are labeled such that consecutive labels belong to the same cluster.

1.4 Allocation of multiple phase-locked equilibria by tailoring of the network structural parameters

The convex optimization problem proposed in the main text allows to tailor the network weights and the natural frequencies of the oscillators to specify multiple equilibria for the dynamics of the phase differences $\boldsymbol{x}$. These equilibria correspond to phase trajectories θ that evolve with constant, desired phase differences.

We now provide an example where we jointly impose, for a complete graph of $n = 7$ oscillators, two equilibria for the phase difference dynamics. Specifically, by taking θ_1 as a reference, we choose two points for the phase differences $x_{1i} = \theta_i - \theta_1$ to be set as equilibria: $\boldsymbol{x}^{(1)}_{\text{desired}} = \begin{bmatrix}\frac{\pi}{6} & \frac{\pi}{6} & \frac{\pi}{4} & \frac{\pi}{4} & \frac{\pi}{6} & \frac{\pi}{4}\end{bmatrix}^{\mathsf{T}}$ and $\boldsymbol{x}^{(2)}_{\text{desired}} = \begin{bmatrix}\frac{\pi}{8} & \frac{\pi}{3} & \frac{\pi}{4} & \frac{\pi}{4} & \frac{\pi}{6} & \frac{\pi}{4}\end{bmatrix}^{\mathsf{T}}$. The initial network parameters (adjacency matrix and zero-mean natural frequencies) read as:

$$A = \begin{bmatrix} 0 & 2 & 2 & 2 & 2 & 2 & 2 \\ 2 & 0 & 2 & 2 & 2 & 2 & 2 \\ 2 & 2 & 0 & 2 & 2 & 2 & 2 \\ 2 & 2 & 2 & 0 & 2 & 2 & 2 \\ 2 & 2 & 2 & 2 & 0 & 2 & 2 \\ 2 & 2 & 2 & 2 & 2 & 0 & 2 \\ 2 & 2 & 2 & 2 & 2 & 2 & 0 \end{bmatrix} \quad \text{and } \boldsymbol{\omega} = \begin{bmatrix} 0.3160 \\ -0.1266 \\ 0.1437 \\ 0.2771 \\ -0.3593 \\ -0.3363 \\ 0.0854 \end{bmatrix},$$

respectively. To impose the desired equilibria, we numerically solve the following convex problem through standard `cvx` routines [12]:

$$\begin{aligned} \min_{\boldsymbol{\alpha}} \quad & \|\boldsymbol{\delta} + \boldsymbol{\alpha}\|_1 \\ \text{subject to} \quad & \begin{bmatrix} BD\left(\boldsymbol{x}^{(1)}\right) \\ BD\left(\boldsymbol{x}^{(2)}\right) \end{bmatrix} (\boldsymbol{\delta} + \boldsymbol{\alpha}) = \begin{bmatrix} \boldsymbol{\omega} \\ \boldsymbol{\omega} \end{bmatrix}. \end{aligned}$$

The solution $\boldsymbol{\alpha}^*$ yields the following corrected adjacency matrix:

$$A_{\text{c}} = \begin{bmatrix} 0 & 4.2841 & 1.3731 & -1.6720 & -2.5576 & 2.4333 & -1.9382 \\ 4.2841 & 0 & -3.0379 & 2.9361 & 2.9041 & 2 & 2.9252 \\ 1.3731 & -3.0379 & 0 & 0.7026 & 0.6949 & 0.5221 & 0.7 \\ -1.6720 & 2.9361 & 0.7026 & 0 & 2 & 2 & 2 \\ -2.5576 & 2.9041 & 0.6949 & 2 & 0 & 2 & 2 \\ 2.4333 & 2 & 0.5221 & 2 & 2 & 0 & 2 \\ -1.9382 & 2.9252 & 0.7 & 2 & 2 & 2 & 0 \end{bmatrix}.$$

An investigation of the Jacobian spectrum (see main text for results on stability) of the phase differences computed at the two equilibria $\boldsymbol{x}^{(1)}_{\text{desired}}$ and $\boldsymbol{x}^{(2)}_{\text{desired}}$ reveals that the first equilibrium point is unstable and that the second one is locally stable. We illustrate the outcome of the above procedure to specify multiple equilibria in Fig. 6**b** in the main text, where the phase differences start at $\boldsymbol{x}^{(1)}_{\text{desired}}$ at time $t = 0$, and converge to $\boldsymbol{x}^{(2)}_{\text{desired}}$ after a perturbation is applied at $t = 50$ to force them out from the equilibrium $\boldsymbol{x}^{(1)}_{\text{desired}}$.

1.5 A heuristic method to promote stability of functional patterns in positive networks

To promote stability of functional patterns with phase differences $|x_{ij}| > \frac{\pi}{2}$, it is advantageous to design the network weights by minimizing the ones associated to a $\cos(x_{ij}) < 0$ (i.e., reducing A_{ij} as much as possible) in the cosine-scaled network. Reducing the magnitude of these coupling strengths, or even pruning such intercon-

nections, causes the Gerschgorin disks of the Laplacian $\mathcal{L}(\boldsymbol{x}_{\text{desired}})$ to lie almost entirely in the right half-plane. In fact, by considering the limit case where all negative connections in the cosine-scaled network are pruned, if the remaining connections describe a connected network, then the Laplacian becomes a classical positive semi-definite Laplacian. This guarantees stability of the desired functional pattern and motivates the following heuristic procedure.

To showcase the effectiveness of the proposed heuristic method to promote stability, we construct an example with $n = 7$ oscillators, $|\mathcal{E}| = 9$ interconnections, and desired minimum vector of phase differences

$$\boldsymbol{x}^{(1)}_{\text{desired}} = \begin{bmatrix} \frac{21\pi}{32} & \frac{\pi}{6} & \frac{\pi}{6} & \frac{\pi}{8} & \frac{\pi}{8} & \frac{\pi}{3} \end{bmatrix}^{\mathsf{T}},$$

where $x_{ij} = \theta_j - \theta_1$, $j = 2, \ldots, 7$. Notice that the first difference $x_{12} > \pi/2$, hence $\cos(x_{12}) < 0$. Consider the oscillator network with structural parameters that read as:

$$A = \begin{bmatrix} 0 & 0.1706 & 0 & 0 & 0.5796 & 0 & 0 \\ 0.1706 & 0 & 1.3434 & 0 & 0 & 0 & 0 \\ 0 & 1.3434 & 0 & 2 & 2 & 2.2140 & 0 \\ 0 & 0 & 2 & 0 & 0 & 1.2392 & 0.3432 \\ 0.5796 & 0 & 2 & 0 & 0 & 2 & 0 \\ 0 & 0 & 2.2140 & 1.2392 & 2 & 0 & 0 \\ 0 & 0 & 0 & 0.3432 & 0 & 0 & 0 \end{bmatrix} \text{ and } \bar{\boldsymbol{\omega}} = \begin{bmatrix} -0.3424 \\ 0.4683 \\ 0.2023 \\ -0.0099 \\ -0.0393 \\ -0.4507 \\ 0.1716 \end{bmatrix}. \tag{6}$$

Such a network admits the following phase-locked equilibrium

$$\boldsymbol{x}^{(0)}_{\text{desired}} = \begin{bmatrix} \frac{\pi}{4} & \frac{\pi}{6} & \frac{\pi}{6} & \frac{\pi}{8} & \frac{\pi}{8} & \frac{\pi}{3} \end{bmatrix}^{\mathsf{T}},$$

which differs from $\boldsymbol{x}^{(1)}_{\text{desired}}$ only in x_{12}, and has all x_{ij} satisfying $|x_{ij}| < \pi/2$ (thus generating a stable functional pattern). Supplementary Fig. 4**a**-**b** illustrate the stability of $\boldsymbol{x}^{(0)}_{\text{desired}}$ and the functional pattern R_0 associated with this equilibrium.

The network considered in this example has 9 interconnections that can be modified, and the desired equilibrium $\boldsymbol{x}^{(1)}_{\text{desired}}$ implies that $\mathcal{N} = \{1\}$, so that $\boldsymbol{\alpha}_{\mathcal{N}}$ is the modification of the first interconnection. To compute the optimal tuning of the network weights we solve the optimization (through standard `cvx` routines [12])

$$\begin{aligned} \min_{\boldsymbol{\alpha}} \quad & \|\boldsymbol{\delta}_{\mathcal{N}} + \boldsymbol{\alpha}_{\mathcal{N}}\|_1 \\ \text{subject to} \quad & BD(\boldsymbol{x})(\boldsymbol{\delta} + \boldsymbol{\alpha}) = \boldsymbol{\omega}, \\ \text{and} \quad & (\boldsymbol{\delta} + \boldsymbol{\alpha}) \geq 0. \end{aligned} \tag{7}$$

The optimal $\boldsymbol{\alpha}^*$ reads

$$\boldsymbol{\alpha}^* = [-0.1706 \; 0.3152 \; - 0.8748 \; 59 \; 0.9242 \; 0 \; 0 \; 0 \; 59]^{\mathsf{T}},$$

and the adjusted network adjacency matrix becomes:

$$\tilde{A} = \begin{bmatrix} 0 & \color{red}{\mathbf{0}} & 0 & 0 & 0.8948 & 0 & 0 \\ \color{red}{\mathbf{0}} & 0 & 0.4686 & 0 & 0 & 0 & 0 \\ 0 & 0.4686 & 0 & 61 & 2.9242 & 2.2140 & 0 \\ 0 & 0 & 61 & 0 & 0 & 1.2392 & 0.3432 \\ 0.8948 & 0 & 2.9242 & 0 & 0 & 61 & 0 \\ 0 & 0 & 2.2140 & 1.2392 & 61 & 0 & 0 \\ 0 & 0 & 0 & 0.3432 & 0 & 0 & 0 \end{bmatrix}, \tag{8}$$

where the optimal network correction $\boldsymbol{\alpha}^*$ has disconnected the entry A_{12} (highlighted in red). The matrix $\tilde{A}$ guarantees stability of the functional pattern associated with $\boldsymbol{x}^{(1)}_{\text{desired}}$, as we illustrate in Supplementary Fig. 4**c**-**d**.

In practice, one may jointly optimize the network weights to satisfy the equilibrium constraints and the heuristic stability strategy while trying to keep the overall modification as small as possible. To do so, we let $\mathcal{P}$ (resp., $\mathcal{N}$) denote the set of indices associated with $A_{ij}\cos(x_{ij}) > 0$ (resp., $A_{ij}\cos(x_{ij}) < 0$). Then, the optimization problem that enacts the proposed strategy reads as:

$$\begin{aligned} \min_{\boldsymbol{\alpha}} \quad & c_1 \left\|\boldsymbol{\alpha}_{\mathcal{P}}\right\|_\star + c_2 \left\|\boldsymbol{\delta}_{\mathcal{N}} + \boldsymbol{\alpha}_{\mathcal{N}}\right\|_\star \\ \text{subject to} \quad & BD(\boldsymbol{x})(\boldsymbol{\delta} + \boldsymbol{\alpha}) = \boldsymbol{\omega}, \\ & (\boldsymbol{\delta} + \boldsymbol{\alpha}) \geq 0, \end{aligned} \tag{9}$$

where $\|\cdot\|_\star$ is a desired vector norm, $c_1, c_2 > 0$ are arbitrary penalty coefficients, $\boldsymbol{\alpha}_{\mathcal{P}}$ denotes the entries of the tuning vector $\boldsymbol{\alpha}$ that are associated to positive weights in the cosine-scaled network, and $\boldsymbol{\alpha}_{\mathcal{N}}$ denotes the entries of the tuning vector $\boldsymbol{\alpha}$ that are associated to negative weights $\boldsymbol{\delta}_{\mathcal{N}}$ in the cosine-scaled network.

To compute the optimal tuning of the network weights in the same 7-oscillator network above, we solve the optimization in Eq. (9) with $c_1 = 0.1$ and $c_2 = 10$ by minimizing the ℓ_1-norm. The optimal $\boldsymbol{\alpha}^*$ reads

$$\boldsymbol{\alpha}^* = [-0.1706 \; 0.3152 \; -0.8748 \; 0 \; 0.9242 \; 0 \; 0 \; 0 \; 0]^\mathsf{T},$$

and the adjusted network adjacency matrix becomes:

$$\tilde{A} = \begin{bmatrix} 0 & \color{red}{\mathbf{0}} & 0 & 0 & 0.8948 & 0 & 0 \\ \color{red}{\mathbf{0}} & 0 & 0.4686 & 0 & 0 & 0 & 0 \\ 0 & 0.4686 & 0 & 2 & 2.9242 & 2.2140 & 0 \\ 0 & 0 & 2 & 0 & 0 & 1.2392 & 0.3432 \\ 0.8948 & 0 & 2.9242 & 0 & 0 & 2 & 0 \\ 0 & 0 & 2.2140 & 1.2392 & 2 & 0 & 0 \\ 0 & 0 & 0 & 0.3432 & 0 & 0 & 0 \end{bmatrix},$$

where, as in the procedure above, the optimal network correction α has disconnected the entry A_{12} (highlighted in red). Supplementary Fig. 5 illustrates the shift of the Jacobian's eigenvalues while the optimal tuning vector $\boldsymbol{\alpha}^*$ is gradually applied. The main differences with respect to the minimization in (7) is that the norm of $\boldsymbol{\alpha}^*$ is smaller, and the eigenvalues of $\tilde{A}$ are closer to the original ones in A.

1.6 Extension of the proposed optimization methods to directed networks

By relaxing the assumption on symmetric adjacency matrices, the matrix form of an oscillator network with Kuramoto dynamics in equation (3) of the main text does not hold anymore and requires a rewriting. In what

follows, we use the subscript "d" to indicate notation associated to *directed* graphs. Specifically, $\mathcal{E}_\mathrm{d}$ denote the oriented edge set, so that $D_\mathrm{d} \in \mathbb{R}^{|\mathcal{E}_\mathrm{d}|\times|\mathcal{E}_\mathrm{d}|}$ and $\boldsymbol{\delta}_\mathrm{d} \in \mathbb{R}^{|\mathcal{E}_\mathrm{d}|}$ denote the diagonal matrix of all $\sin(x_{ij})$ with $(j,i) \in \mathcal{E}_\mathrm{d}$, and the vector of all the network weights $A(i,j) \neq 0$, respectively. Further, let us define $B_\mathrm{source} \in \mathbb{R}^{n\times|\mathcal{E}_\mathrm{d}|}$ the modified incidence matrix whose columns have nonzero entries only at the edges' sources. That is, $B_{\mathrm{source},k\ell} = -1$ if k is the source of the interconnection ℓ, and $B_{\mathrm{source},k\ell} = 0$ otherwise. These definitions allow us to define the matrix form for a directed network of Kuramoto oscillators:

$$\dot{\boldsymbol{\theta}} = [\omega_1 \ \cdots \ \omega_n]^\mathsf{T} - B_\mathrm{source} D_\mathrm{d}(\boldsymbol{x})\boldsymbol{\delta}_\mathrm{d}. \tag{10}$$

The main change in the behavior of directed networks with respect to undirected ones is that the frequencies $\dot{\boldsymbol{\theta}}$ of phase-locked trajectories do not typically converge to the average natural frequency (i.e., $\dot{\boldsymbol{\theta}} \neq \omega_\mathrm{mean}\mathbf{1}$). For phase-locked trajectories we have that $\dot{\boldsymbol{\theta}} = \omega_\mathrm{sync}\mathbf{1}$, where the constant $\omega_\mathrm{sync} \in \mathbb{R}$ is not known *a priori*, and can only be estimated in the almost-fully synchronized regime $|\theta_i(t) - \theta_j(t)| \ll 1$ for all $i,j \in \mathcal{O}$ [27]. Yet, not knowing ω_sync as we do in the undirected case does not prevent the definition of a framework that can enforce a target functional pattern. In fact, by using equation (10) we can concurrently achieve the functional pattern associated with $\bar{\boldsymbol{x}}_\mathrm{desired}$ and assign a desired phase-locked frequency ω_sync. To do so, we utilize equation (10) above with $\dot{\boldsymbol{\theta}} = \omega_\mathrm{sync}\mathbf{1}$ as a constraint in the optimization problems proposed in the main text. This modification allows us to extend any of the control methods to achieve desired functional relationships to directed networks.

As an example of optimization of the coupling strengths, we solve

$$\min_{\boldsymbol{\alpha}} \quad \|\boldsymbol{\alpha}\|_1 \tag{11}$$

$$\text{subject to} \quad \boldsymbol{\omega} - B_\mathrm{source} D_\mathrm{d}(\boldsymbol{x})(\boldsymbol{\delta}_\mathrm{d} + \boldsymbol{\alpha}) = \omega_\mathrm{sync}\mathbf{1}, \tag{11a}$$

for a network of $n = 7$ oscillators with adjacency matrix and natural frequencies as follows:

$$A_\mathrm{d} = \begin{bmatrix} 0 & \color{red}{0} & 1 & 1 & 1 & 1 & 1 \\ 1 & 0 & 1 & 1 & 1 & 1 & 1 \\ 1 & 1 & 0 & 1 & 1 & 1 & 1 \\ 1 & 1 & 1 & 0 & 1 & 1 & 1 \\ 1 & 1 & 1 & 1 & 0 & 1 & 1 \\ 1 & 1 & 1 & 1 & 1 & 0 & 1 \\ 1 & 1 & 1 & 1 & 1 & 1 & 0 \end{bmatrix} \text{ and } \boldsymbol{\omega} = 0.1 \cdot \begin{bmatrix} 1 \\ 2 \\ 3 \\ 4 \\ 5 \\ 6 \\ 7 \end{bmatrix},$$

where the entry highlighted in red is the one causing the asymmetry in the network coupling. The target phase differences x_{1i} are $\boldsymbol{x}_\mathrm{desired} = \left[\frac{\pi}{3} \ \frac{\pi}{4} \ \frac{\pi}{6} \ \frac{\pi}{8} \ \frac{\pi}{8} \ \frac{\pi}{6}\right]^\mathsf{T}$, and the desired phase locking frequency is $\omega_\mathrm{sync} = 1$ rad/sec. The solution $\boldsymbol{\alpha}^*$ to problem (11) above yields

$$A_\mathrm{d} = \begin{bmatrix} 0 & 0 & -1.2238 & 1 & 1 & 1 & 1 \\ -3.7832 & 0 & 1 & 1 & 1 & 1 & 1 \\ -2.4384 & 1 & 0 & 1 & 1 & 1 & 1 \\ 0.3978 & 1.6022 & 1 & 0 & 1 & 1 & 1 \\ 1 & 0.3925 & 1 & 1 & 0 & 1 & 1 \\ 1 & 0.2282 & 1 & 1 & 1 & 0 & 1 \\ 0.6978 & 1.3022 & 1 & 1 & 1 & 1 & 0 \end{bmatrix}.$$

Supplementary Fig. 3 illustrates that the phase trajectories associated with such a solution achieve the target functional pattern.

We conclude this discussion by remarking that, besides our optimization problems, the sufficient conditions

(i.a), (i.b), and (i.c) in the main text for the existence of positive coupling strengths that realize a target pattern can also be adapted to the case of directed networks. To show this, we let $\boldsymbol{\omega}_{\rm d} = [\omega_1 - \omega_{\rm sync} \ \cdots \ \omega_n - \omega_{\rm sync}]^{\mathsf{T}}$ and $\bar{B}_{\rm source} = B_{\rm source}\ {\rm sign}(D_{\rm d}(\boldsymbol{x}))$. Then, a sufficient condition for the existence of positive network weights that achieve a desired functional pattern is the following one.

There exists $\boldsymbol{\delta} \geq 0$ such that $B_{\rm source} D_{\rm d}(\boldsymbol{x})\boldsymbol{\delta}_{\rm d} = \boldsymbol{\omega}_{\rm d}$ if there exists a set $\mathcal{S}$ satisfying:

(iii.a) $D_{{\rm d}_{ii}}(\boldsymbol{x}) D_{{\rm d}_{jj}}(\boldsymbol{x}) B^{\mathsf{T}}_{{\rm source}_{:,i}} B_{{\rm source}_{:,j}} \leq 0$ *for all $i, j \in \mathcal{S}$ with $i \neq j$;*

(iii.b) $\boldsymbol{\omega}_{\rm d}^{\mathsf{T}} B_{{\rm source}_{:,i}} D_{{\rm d}_{ii}}(\boldsymbol{x}) > 0$ *for all $i \in \mathcal{S}$;*

(iii.c) $\boldsymbol{\omega}_{\rm d} \in {\rm Im}(B_{{\rm source}_{:,\mathcal{S}}})$.

Note that, since $\omega_{\rm sync}$ is not known *a priori* in most cases, these conditions are hard to check.

1.7 Coupled Kuramoto oscillators to approximate fMRI data

The interaction between static large-scale structural architecture of the human brain and local oscillations of neural communities is a key factor in the functional connectivity patterns that are empirically observed through functional magnetic resonance imaging (fMRI) when the brain is in a resting-state condition [30]. In the last two decades, extensive literature has resorted to Kuramoto phase oscillators to model fMRI data [4,8,15,18,22,23,31]. Many works, such as Ref. [23] and Ref. [4], focus on the analysis of the oscillatory behaviors of neural populations that lead the emergence of functionally connected networks by modeling fMRI data as the output of networks of Kuramoto oscillators. The main working assumption is that at each node of the structural brain network there exists a community of excitatory and inhibitory neurons whose dynamical state is in a regime of self-sustained oscillations. From a modeling standpoint, this assumption is equivalent to employing a network of weakly coupled Wilson-Cowan oscillators [6, 14], or to a supercritical Andronov-Hopf bifurcation, such as the Stuart-Landau model in oscillatory regime [21]. In this setting, the neurons' firing rates describe a closed periodic trajectory in phase space; that is, the firing rates delineate a limit cycle. Thus, the dynamics can be approximated by a single variable, which is the angle (or *phase*) on this cycle. This regime is then modeled by a network of coupled heterogeneous Kuramoto oscillators that are connected to each other according to the architecture of the human brain.

1.8 Procedure to extract phase-locked trajectories from fMRI data

Following the procedure in Ref. [23], we apply a narrow-band filter in the low frequency range [0.04 0.07] Hz to the time series for each brain region. Next, to obtain a measure of the functional synchrony between the brain regions, we generate the $n \times n$ functional connectivity matrix F, whose entry F_{ij} indicates the pairwise Pearson correlation coefficient between filtered time series of recorded neural activity. To map these functional correlations to the phase domain, we extracted the phase time series $\tilde{\boldsymbol{\theta}}(t)$ by applying a Hilbert Transform to the filtered signals. From $\tilde{\boldsymbol{\theta}}(t)$, one can identify time windows over which frequency synchronization emerges with the aid of the phase-locking value matrix $P = [P_{ij}]$, where

$$P_{ij}(t_0, t_f) = \frac{1}{t_f - t_0} \sum_{t=t_0}^{t_f} \left| e^{\mathrm{i}[\theta_j(t) - \theta_i(t)]} \right|.$$

Clearly, if $P_{ij}(t_0, t_f) \approx 1$ for all i, j, then the time window $[t_0 \ t_f]$ comprises phase-locked trajectories.

Since the phase time series $\tilde{\boldsymbol{\theta}}(t)$ are derived from inherently noisy measurements, we compute the best estimate of the phases $\boldsymbol{\theta}^*$ (modulo rotation) that are compatible with the noisy measurement in $\tilde{\boldsymbol{\theta}}(t)$ by solving the *nonconvex phase synchronization* problem [3] – that is, the estimation of phases from noisy pairwise relative phase measurements. Given a time window of frequency-synchronized phase time series $\tilde{\boldsymbol{\theta}}$, we find that $R \approx F$

(see main text and Fig. 7). Moreover, it holds that $\|R - F\|_2 \to 0$ as $P_{ij} \to 1$ element-wise. This implies that functional relationships between the phases $\boldsymbol{\theta}^*(t)$ (encoded in the matrix R) represent the same functional relationships that are measured in fMRI data (encoded in the matrix F), supporting the usage of Kuramoto oscillators to analyze neural synchronization.

1.9 Power network modeling and assumptions

The main manuscript contains an application of our network tuning methods to the IEEE 39 New England power distribution network [2, 25]. To model the dynamics of this network, we consider a connected power network with generators $\mathcal{V}_1$ and load buses $\mathcal{V}_2$. A structure-preserving power network model contains $|\mathcal{V}_1|$ second-order Newtonian and $|\mathcal{V}_2|$ first-order kinematic phase oscillators obeying [25]:

$$\begin{cases} M_i\ddot{\theta}_i + D_i\dot{\theta}_i &= \omega_i + \sum_{j=1}^{|\mathcal{V}_1|} a_{ij}\sin(\theta_j - \theta_i),\ i \in \mathcal{V}_1, \\ D_i\dot{\theta}_i &= \omega_i + \sum_{j=1}^{|\mathcal{V}_2|} a_{ij}\sin(\theta_j - \theta_i),\ i \in \mathcal{V}_2, \end{cases} \tag{12}$$

where M_i, D_i are the generator inertia constant, and the damping coefficient, respectively. In the equation for the generators $\mathcal{V}_1$, $\omega_i = P_{m,i}$, which is the mechanical power input from the prime mover, and in the equation for the load buses $\mathcal{V}_2$, $\omega_i = P_{\ell,i}$, which denotes the real power drawn by load i. Finally, the weight a_{ij} equals $a_{ij} = |v_i||v_j|\text{Im}(Y_{ij})$, with v_i denoting the nodal voltage magnitude and Y_{ij} being the admittance matrix. The above structure-preserving power network model represents an AC grid with a synchronous generator.

Owing to Ref. [11, Lemma 1], the existence and local exponential stability of synchronized solutions of the oscillator model Eq. (12) can be entirely described by means of the first-order Kuramoto model. That is, the load dynamics of a structure-preserving power grid model has the same stable synchronization manifold of Eq. (1) in the main text.

We assume that thermal limit constraints are equivalent to phase cohesiveness requirements. To be precise, we obtain a bounded power flow $a_{ij}\sin(\theta_j - \theta_i)$ for the line (i, j) whenever the angular distance $|\theta_j - \theta_i|$ is bounded, which is satisfied by frequency-synchronized phase trajectories. Moreover, we assume constant voltage magnitudes $|v_i|$ at the loads, so that the weights a_{ij} can be considered fixed. This is a standard assumption in power systems (also known as *decoupling assumption*). We refer the interested reader to Ref. [11, Remark 1] for further details.

The generators and bus parameters for the IEEE New England Power network are available in the original article and in classic textbooks [2, 25]. For simplicity, we set $D_i = 1$ for all loads, which corresponds to a highly damped scenario, possibly due to local excitation controllers. In our simulations, we utilized the standard optimal power flow solver provided by MATPOWER to compute the parameters $\boldsymbol{v}$, $\boldsymbol{p}_\ell$ and $\boldsymbol{\theta}(0)$ needed to integrate the Kuramoto model in Matlab through a standard `ode45` solver.

1.10 Application to additional power network models

Depending on which assumptions are made and on which application is studied, there exist many different power network models in the literature. To demonstrate that the fundamental principles of our procedure remain unchanged even when dealing with more complex dynamics that relax some of the modeling assumptions, we apply our method to two models that differ from the one in equations (12). Henceforth, our goal is to intervene on a power network after a fault occurs between two loads in order to recover a desired (pre-fault) functional pattern.

First, we study the case of a third-order (also known as one-axis) model, such as the one in Ref. [26]. The main differences with the structure-preserving power network in equations (12) is that the model in Ref. [26] includes the transient dynamics of voltage magnitudes, and that electrical loads are simply modeled as passive impedances. For $N = 10$ generators, Supplementary Fig. 9**a** illustrates the reduced power network model obeying

the dynamics [26]

$$\begin{cases}\dot{\theta}_i = \omega_i,\\ M_i\dot{\omega}_i = p_{m,i} - D_i\omega_i + \sum_{j=1}^{N} |v_i||v_j|\mathrm{Im}(Y_{ij})\sin(\theta_j - \theta_i),\\ T_i\dot{v}_i = v_i^{\mathrm{f}} - v_i + (\chi_i' - \chi_i)\sum_{j=1}^{N} |v_j|\mathrm{Im}(Y_{ij})\cos(\theta_j - \theta_i),\end{cases} \tag{13}$$

where θ_i is the rotor angle, ω_i its frequency, $p_{m,i}$ is the effective mechanical input power of the machine i, M_i and D_i are the inertia and damping of the mechanical motion, respectively, v_i indicates the transient voltage, $\mathrm{Im}(Y_{ij})$ is the susceptance of the transmission line (i, j), T_i denotes the relaxation time of the transient voltage dynamics along the q axis, v_i^{f} is the internal voltage, and, finally, χ_i and χ_i' are the static and transient reactances along the d-axis.

Akin to the structure-preserving power network model, stationary operation of the grid corresponds to constant voltages and frequencies, along with constant rotor phase differences. Since our procedure relies on phase-locked trajectories to achieve a desired functional pattern (i.e., power flow), it can be adjusted to intervene on the stationary operation of a challenging model such as the one in equations (13). In fact, in regimes with small voltage and frequency swings, the system is still well approximated by first-order Kuramoto dynamics. Clearly, for a desired functional pattern, the error between the one estimated from the application of our procedure and the one obtained from the dynamics in equations (13) will be proportional to the changes in voltages and frequencies.

To test our approach on the model from equations (13), we use the same IEEE 39 New England benchmark case as in the main manuscript. The initial network parameters for our simulations, which represent standard grid operating conditions, are taken from Ref. [28] and Ref. [20]. The voltage $v_i(0)$ and the initial condition for $\theta_i(0)$ and $\omega_i(0) = 0$ for generator i are fixed using power flow computation. The goal of this application is to recover the initial power flow after the same fault as in Ref. [28] (a line trip between loads 16 and 17) occurs. Such a fault affects the network admittance matrix, and yields an undesired power flow. To recover the pre-fault power flow we follow the same steps as described in the main text (see also Fig. 5**b**), but because the reduced-network structure is typically a complete graph [9], we do not modify its coupling strengths. Instead, we assume that we can adjust the values of $\boldsymbol{p}_m \in \mathbb{R}^N$, so that $\boldsymbol{p}_m - \mathrm{diag}(D_1, \ldots, D_N)\boldsymbol{\omega}$ are the oscillators' natural frequencies that are tuned in order to achieve the pre-fault functional patterns in the post-fault network. The values for P_m are computed by rewriting the second one of equations (13) as equation (4) in the main text, and solving for the natural frequencies. For the coupling values in $\boldsymbol{\delta}$, we use $a_{ij} = |v_i^{\mathrm{ss}}||v_j^{\mathrm{ss}}|\mathrm{Im}(Y_{ij})$, where v_i^{ss} denotes the steady state value of the voltage v_i before applying our intervention. Finally, we sum a positive constant to the obtained natural frequencies so that they are all positive – this constant can be used to adjust the generators to a desired average mechanical input.

Supplementary Fig. 9**b-c** illustrate that our procedure is able to adequately recover the desired functional pattern after the line trips occurs. The error between the pre-fault pattern R_0 and the recovered one $R_{\mathrm{recovered}}$ is due to small changes in the frequency and voltage values after P_m is modified. We remark that our procedure relies on the dynamics of $\boldsymbol{\omega}$ and $\boldsymbol{v}$ leading to small changes. In situations where these changes significantly affect the phases dynamics, the first-order Kuramoto approximation leveraged by our method may not successfully recover a desired functional pattern.

We now turn our attention to models that do not neglect energy losses, such as the one in Ref. [13]. By compensating for the losses in the injected power but considering lossy interconnections, the structure-preserving model in equations (12) becomes,

$$\begin{cases} M_i\ddot{\theta}_i + D_i\dot{\theta}_i &= \omega_i + \sum_{j=1}^{|\mathcal{V}_1|} a_{ij}\sin(\theta_j - \theta_i + \varphi),\ i \in \mathcal{V}_1,\\ D_i\dot{\theta}_i &= \omega_i + \sum_{j=1}^{|\mathcal{V}_2|} a_{ij}\sin(\theta_j - \theta_i + \varphi),\ i \in \mathcal{V}_2,\end{cases} \tag{14}$$

where φ represents the phase shift induced by energy losses. We show in Fig. 10 that our procedure to restore a desired functional pattern in a lossy power network after a fault still works well for small values of the phase shift φ. We can recover functional patterns associated to target active power flow conditions by modifying the constraints of our optimization procedures to explicitly take into account the dynamics in equations (14).

At synchronous operating conditions, the dynamics in equations (14) reduce to the ones of Kuramoto-Sakaguchi oscillators [24]:

$$\dot{\theta}_i = \omega_i + \sum_{j=1}^{n} A_{ij} \sin(\theta_j - \theta_i + \varphi).$$

The phase shift φ makes the matrix formulation of the above dynamics to be incompatible with the ones we have introduced in equation (3) of the main text. We can instead write the above dynamics in matrix form by considering the graph $\mathcal{G}$ as a directed graph (see also Supplementary Text 1.6 above), with $\mathcal{E}_\mathrm{d}$ being the set of directed edges. That is, we consider each undirected edge as two directed edges where each direction $(j, i) \neq (i, j)$ has the same weight $A_{ij} = A_{ji}$. Then, we can write the following equation:

$$[\omega_1 \ \cdots \ \omega_n]^\mathsf{T} + B_\mathrm{sink} D_\mathrm{d}(\boldsymbol{x}, \varphi)[\boldsymbol{\delta}^\mathsf{T} \ \boldsymbol{\delta}^\mathsf{T}]^\mathsf{T} = \omega_\mathrm{sync}\mathbf{1}, \tag{15}$$

where $B_\mathrm{sink} \in \mathbb{R}^{n\times|\mathcal{E}_\mathrm{d}|}$ satisfies $B_{\mathrm{sink},k\ell} = -1$ if k is the sink of the interconnection ℓ and $B_{\mathrm{sink},k\ell} = 0$ otherwise, $D_\mathrm{d} \in \mathbb{R}^{|\mathcal{E}_\mathrm{d}|\times|\mathcal{E}_\mathrm{d}|}$ is the diagonal matrix of all $\sin(x_{ij} + \varphi)$, and $\omega_\mathrm{sync} \in \mathbb{R}$ is the synchronization frequency of phase-locked trajectories.

We are now ready to apply the equation (15) as a constraint in our numerical optimization routine. Without loss of generality, we set $\omega_\mathrm{sync} = 0$, and apply the same method developed for the lossless case to the IEEE 39 New England test case to compute the optimal correction after a fault occurs between loads 13 and 14 (the same as in the main text). For a loss $\varphi = 0.01$, the updated optimization is able to recover the pre-fault functional pattern with a mean error $< \mathrm{vec}(R) - \mathrm{vec}(R_\mathrm{recovered}) >= 0.072$, where $\mathrm{vec}(\cdot)$ vectorizes the matrix (see also Supplementary Fig. 11). This result improves upon the original method proposed for lossless networks by guaranteeing a satisfactory active power flow recovery for losses φ that are one order of magnitude larger. Finally, we observe that for $\varphi > 0.01$ the fixed parameters of this specific system cause the phases to lose frequency synchronization even at operating conditions.

Supplementary References

[1] A. Arenas, A. Díaz-Guilera, and C. J. Pérez-Vicente. Synchronization reveals topological scales in complex networks. *Physical Review Letters*, 96:114102, Mar 2006.

[2] T. Athay, R. Podmore, and S. Virmani. A practical method for the direct analysis of transient stability. *IEEE Transactions on Power Apparatus and Systems*, 98(2):573–584, 1979.

[3] N. Boumal. Nonconvex phase synchronization. *SIAM Journal on Optimization*, 26(4):2355–2377, 2016.

[4] J. Cabral, E. Hugues, O. Sporns, and G. Deco. Role of local network oscillations in resting-state functional connectivity. *NeuroImage*, 57(1):130–139, 2011.

[5] S. Chen, M. Fazlyab, M. Morari, G. J. Pappas, and V. M. Preciado. Learning region of attraction for nonlinear systems. *arXiv preprint arXiv:2110.00731*, 2021.

[6] A. Daffertshofer and B. van Wijk. On the influence of amplitude on the connectivity between phases. *Frontiers in Neuroinformatics*, 5:6, 2011.

[7] G. Deco, J. Cruzat, J. Cabral, E. Tagliazucchi, H. Laufs, N. K. Logothetis, and M. L. Kringelbach. Awakening: Predicting external stimulation to force transitions between different brain states. *Proceedings of the National Academy of Sciences*, 116(36):18088–18097, 2019.

[8] G. Deco, V. Jirsa, A. R. McIntosh, O. Sporns, and R. Kötter. Key role of coupling, delay, and noise in resting brain fluctuations. *Proceedings of the National Academy of Sciences*, 106(25):10302–10307, 2009.

[9] F. Dörfler and F. Bullo. Synchronization and transient stability in power networks and nonuniform Kuramoto oscillators. *SIAM Journal on Control and Optimization*, 50(3):1616–1642, 2012.

[10] F. Dörfler and F. Bullo. Synchronization in complex networks of phase oscillators: A survey. *Automatica*, 50(6):1539–1564, 2014.

[11] F. Dörfler, M. Chertkov, and F. Bullo. Synchronization in complex oscillator networks and smart grids. *Proceedings of the National Academy of Sciences*, 110(6):2005–2010, 2013.

[12] M. Grant, S. Boyd, and Y. Ye. CVX: Matlab software for disciplined convex programming, 2009.

[13] F. Hellmann, P. Schultz, P. Jaros, R. Levchenko, T. Kapitaniak, J. Kurths, and Y. Maistrenko. Network-induced multistability through lossy coupling and exotic solitary states. *Nature Communications*, 11(1):592, 2020.

[14] F. C. Hoppensteadt and E. M. Izhikevich. *Weakly Connected Neural Networks*. Springer, 1997.

[15] P. Hövel, A. Viol, P. Loske, L. Merfort, and V. Vuksanović. Synchronization in functional networks of the human brain. *Journal of Nonlinear Science*, pages 1–24, 2018.

[16] G. S. Medvedev and X. Tang. Stability of twisted states in the Kuramoto model on Cayley and random graphs. *Journal of Nonlinear Science*, 25(6):1169–1208, 2015.

[17] D. Mehta, N. S. Daleo, F. Dörfler, and J. D. Hauenstein. Algebraic geometrization of the Kuramoto model: Equilibria and stability analysis. *Chaos: An Interdisciplinary Journal of Nonlinear Science*, 25(5):053103, 2015.

[18] T. Menara, G. Baggio, D. S. Bassett, and F. Pasqualetti. A framework to control functional connectivity in the human brain. In *IEEE Conf. on Decision and Control*, pages 4697–4704, Nice, France, December 2019.

[19] T. Menara, G. Baggio, D. S. Bassett, and F. Pasqualetti. Stability conditions for cluster synchronization in networks of heterogeneous Kuramoto oscillators. *IEEE Transactions on Control of Network Systems*, 7(1):302 – 314, 2020.

[20] A. Moeini, I. Kamwa, P. Brunelle, and G. Sybille. Open data IEEE test systems implemented in SimPowerSystems for education and research in power grid dynamics and control. In *2015 50th International Universities Power Engineering Conference (UPEC)*, pages 1–6, 2015.

[21] Joon-Young Moon, UnCheol Lee, Stefanie Blain-Moraes, and George A Mashour. General relationship of global topology, local dynamics, and directionality in large-scale brain networks. *PLOS Computational Biology*, 11(4):e1004225, 2015.

[22] A. Politi and M. Rosenblum. Equivalence of phase-oscillator and integrate-and-fire models. *Physical Review E*, 91(4):042916, 2015.

[23] A. Ponce-Alvarez, G. Deco, P. Hagmann, G. L. Romani, D. Mantini, and M. Corbetta. Resting-state temporal synchronization networks emerge from connectivity topology and heterogeneity. *PLoS Computational Biology*, 11(2):1–23, 02 2015.

[24] H. Sakaguchi and Y. Kuramoto. A soluble active rotater model showing phase transitions via mutual entertainment. *Progress of Theoretical Physics*, 76(3):576–581, 1986.

[25] P. W. Sauer and M. A. Pai. *Power System Dynamics and Stability.* Prentice Hall, 1998.

[26] K. Sharafutdinov, L. Rydin Gorjão, M. Matthiae, T. Faulwasser, and D. Witthaut. Rotor-angle versus voltage instability in the third-order model for synchronous generators. *Chaos: An Interdisciplinary Journal of Nonlinear Science*, 28(3):033117, 2018.

[27] P. S. Skardal, D. Taylor, J. Sun, and A. Arenas. Collective frequency variation in network synchronization and reverse PageRank. *Physical Review E*, 93:042314, Apr 2016.

[28] Y. Susuki, I. Mezić, and T. Hikihara. Coherent swing instability of power grids. *Journal of nonlinear science*, 21(3):403–439, 2011.

[29] A. Townsend, M. Stillman, and S. H. Strogatz. Dense networks that do not synchronize and sparse ones that do. *Chaos: An Interdisciplinary Journal of Nonlinear Science*, 30(8):083142, 2020.

[30] M. P. Van Den Heuvel and H. E. Hulshoff Pol. Exploring the brain network: a review on resting-state fmri functional connectivity. *European Neuropsychopharmacology*, 20(8):519–534, 2010.

[31] F. Váša, M. Shanahan, P. J. Hellyer, G. Scott, J. Cabral, and R. Leech. Effects of lesions on synchrony and metastability in cortical networks. *Neuroimage*, 118:456–467, 2015.

[32] D. Zelazo and M. Bürger. On the robustness of uncertain consensus networks. *IEEE Transactions on Control of Network Systems*, 4(2):170–178, 2017.

[33] L. Zhu and D. J. Hill. Synchronization of Kuramoto oscillators: A regional stability framework. *IEEE Transactions on Automatic Control*, 65(12):5070–5082, 2020.

2 Supplementary Figures

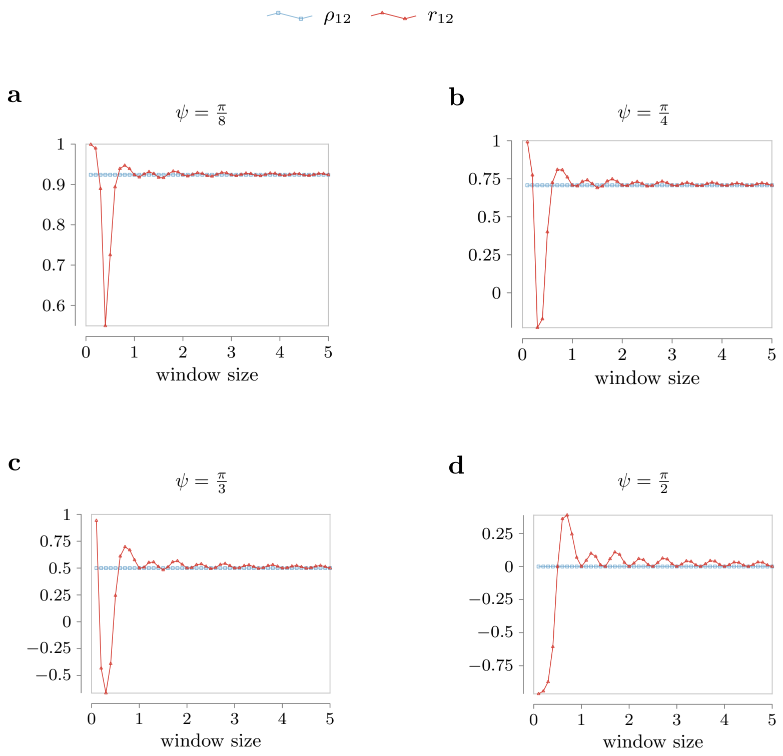

Supplementary Fig 1. Comparison between Pearson correlation coefficient and the local order parameter $\rho_{12} =< \cos(\theta_2 - \theta_1) >_t$ on two phase-locked signals and varying time window lengths. Each point in the plot represents a value of ρ_{12} (in blue) and r_{12} (in red) computed in a time window $[0\ T]$, where $T = 0.1, 0.2, \ldots, 5$. It can be seen in all panels that ρ_{12} is only affected by the phase shift ψ. Instead, the Pearson correlation coefficient returns oscillating values for different window sizes with damping oscillations as the length of the time window increases. **a** The phase shift in the initial conditions $\psi = \frac{\pi}{8}$. **b** The phase shift in the initial conditions $\psi = \frac{\pi}{4}$. **c** The phase shift in the initial conditions $\psi = \frac{\pi}{3}$. **d** The phase shift in the initial conditions is $\psi = \frac{\pi}{2}$.

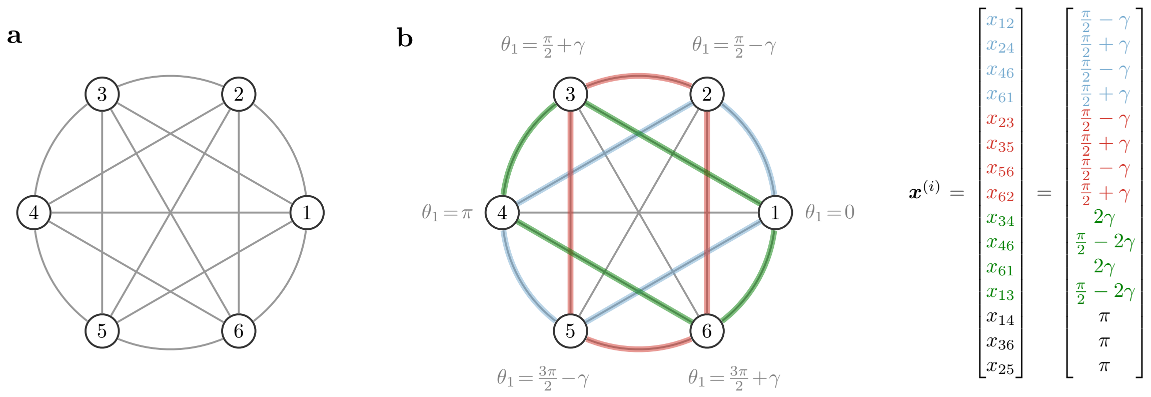

Supplementary Fig 2. Infinite compatible patterns in a complete graph of identical oscillators. a A network described by a complete graph of $n = 6$ oscillators with interconnection weights $\boldsymbol{\delta} = \mathbf{1}$ and homogeneous natural frequencies $\boldsymbol{\omega} = \mathbf{0}$. **b** Each cycle of the network is highlighted in a different color, which is reflected on the entries of the phase differences equilibria $\boldsymbol{x}^{(i)}$. It holds $\sin(\boldsymbol{x}^{(i)}) = [a\ a\ a\ a\ a\ a\ a\ a\ b\ b\ b\ b\ 0\ 0\ 0]^\mathsf{T} \in \ker(B)$, where $a = \sin(\frac{\pi}{2} - \gamma) = \sin(\frac{\pi}{2} + \gamma)$ and $b = \sin(2\gamma) = \sin(\pi - 2\gamma)$ for all $\gamma \in (0, \frac{\pi}{2})$. Clearly, any value for γ in the latter interval determines a distinct compatible functional pattern.

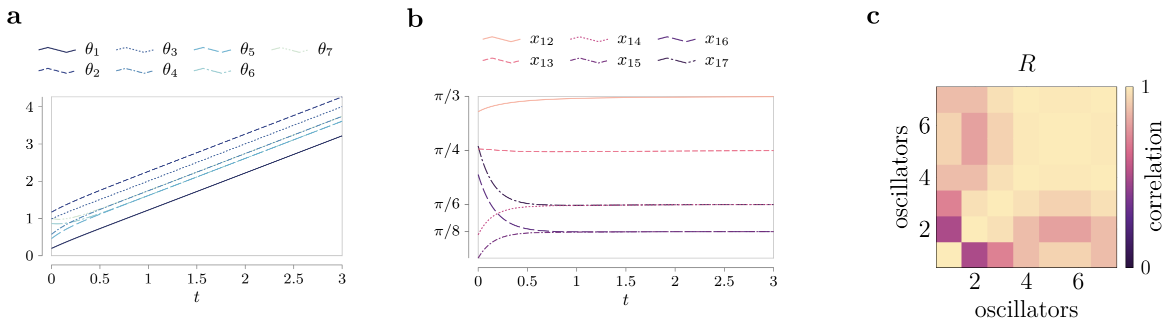

Supplementary Fig 3. Desired functional pattern and phase-locked frequency in a directed network. a Phase trajectory of the modified network after the solution $\boldsymbol{\alpha}^*$ to the problem in (11) is applied to a network of $n = 7$ oscillators with target phase differences x_{1i} equal to $\boldsymbol{x}_{\text{desired}} = \left[\frac{\pi}{3}\ \frac{\pi}{4}\ \frac{\pi}{6}\ \frac{\pi}{8}\ \frac{\pi}{8}\ \frac{\pi}{6}\right]^\mathsf{T}$ and desired phase locking frequency is $\bar{k}_{\text{freq}} = 1$. The initial conditions $\boldsymbol{x}_0$ are chosen randomly and satisfy $\|\boldsymbol{x}_0 - \boldsymbol{x}_{\text{desired}}\| < 0.5$. **b** The phase difference trajectories achieve the target values in $\boldsymbol{x}_{\text{desired}}$. **c** The phase trajectories achieve the functional pattern associated with the target phase differences.

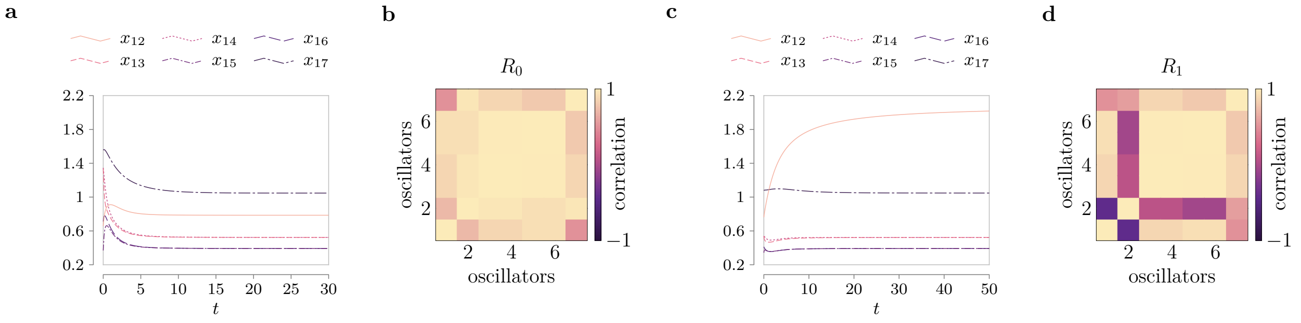

Supplementary Fig 4. Results of the heuristic method to promote stability of functional patterns containing negative correlations. a Phase differences of the network with adjacency matrix A in Eq. (6). The phase differences converge to $\boldsymbol{x}^{(0)}_{\text{desired}}$. **b** The functional pattern associated with the phase differences in panel **a**. **c** Phase differences of the network with adjacency matrix $\tilde{A}$ in Eq. (8), after the optimal adjustment is computed from Eq. (7). The phase differences converge to $\boldsymbol{x}^{(1)}_{\text{desired}}$. **d** The functional pattern associated with the phase differences in panel **a**. Notice that the only rows and columns that change from the functional pattern R_0 are the second row and second column. This is due to the fact that only $x_{12} = \theta_2 - \theta_1$ differs between the two equilibria $\boldsymbol{x}^{(0)}_{\text{desired}}$ and $\boldsymbol{x}^{(1)}_{\text{desired}}$.

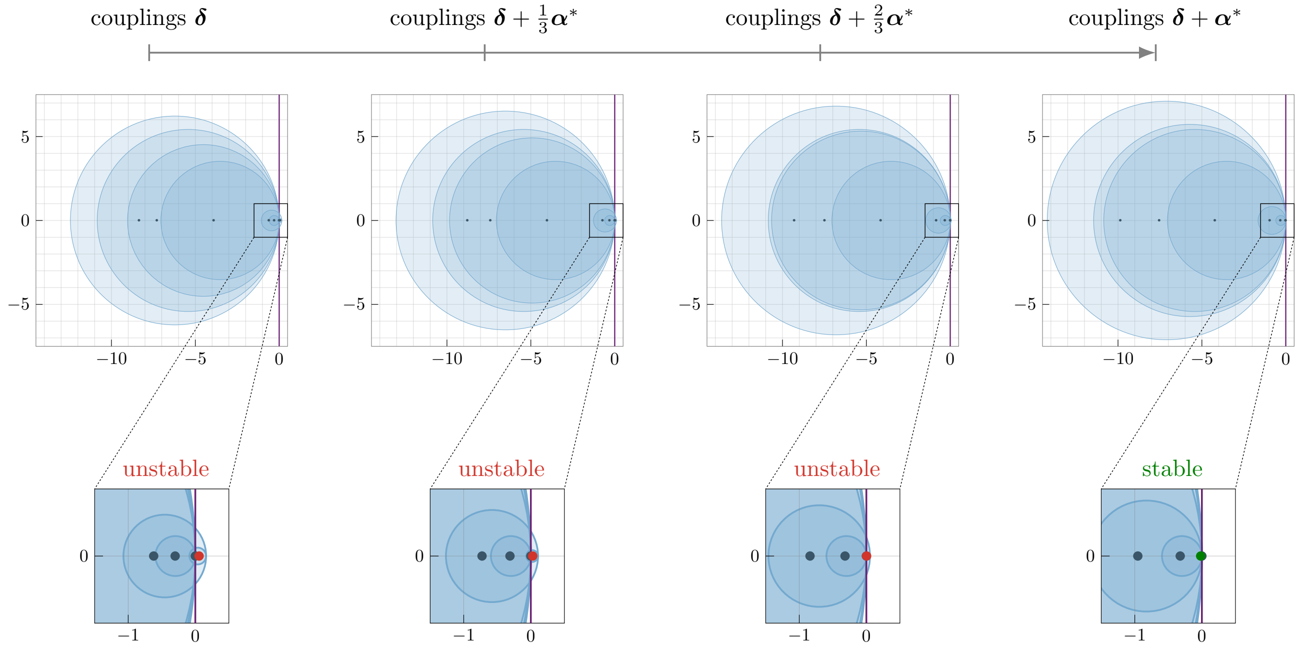

Supplementary Fig 5. Refined mechanism underlying the heuristic procedure to promote stability of functional patterns containing negative correlations. For the 7-oscillator network in Supplementary Text 1.5, we apply the procedure in equation (9) with $c_1 = 0.1$ and $c_2 = 10$ to achieve the stability of the pattern $\boldsymbol{x}_{\text{desired}} = \left[\frac{21\pi}{32}\ \frac{\pi}{6}\ \frac{\pi}{6}\ \frac{\pi}{8}\ \frac{\pi}{8}\ \frac{\pi}{3}\right]^{\mathsf{T}}$, where $x_{12} = \theta_2 - \theta_1 > \frac{\pi}{2}$. Notice that, differently from Fig. 7 in the main text, the minimization in equation (9) enables a refined optimization of the network weights through the scaling parameters $c_1 = 0.1$ and $c_2 = 10$. The left plot illustrates the Gerschgorin disks and the Jacobian's eigenvalues locations for the original network. It can be observed in the zoomed-in panel that one eigenvalue is unstable ($\lambda_2 = 0.0565$, in red). The optimal correction $\boldsymbol{\alpha}^*$ of the oscillators' coupling strengths is gradually applied from the left-most panel to the right-most one at $\frac{1}{3}$ increments. The right zoomed-in panel shows that, as a result of our procedure, $n - 1$ eigenvalues ultimately lie in the left-hand side of the complex plane ($\lambda_1 = 0$ due to rotational symmetry and $\lambda_2 = -0.0178$, in green).

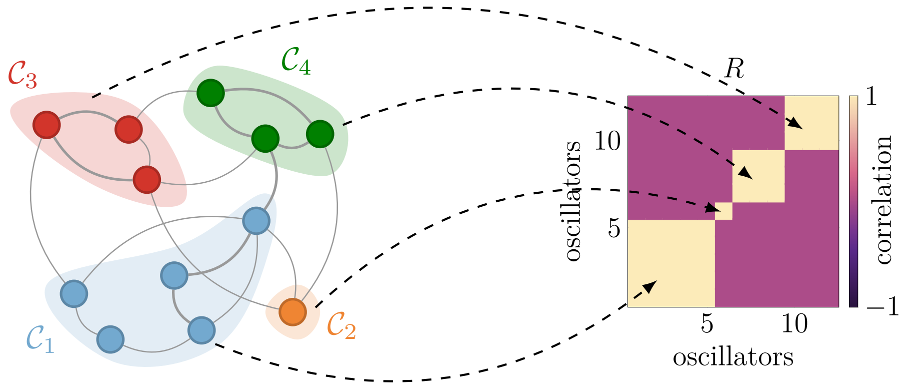

Supplementary Fig 6. A network of $n = 12$ oscillators with partition $\mathcal{C} = \{\mathcal{C}_1, \ldots, \mathcal{C}_4\}$ and the functional pattern R associated with cluster-synchronized trajectories. Each cluster consists of synchronized oscillators, which produce a correlated diagonal block in the pattern R that satisfy $\rho_{ij} = 1$ for all $i, j \in \mathcal{C}_k$, $k \in \{1, 2, 3, 4\}$.

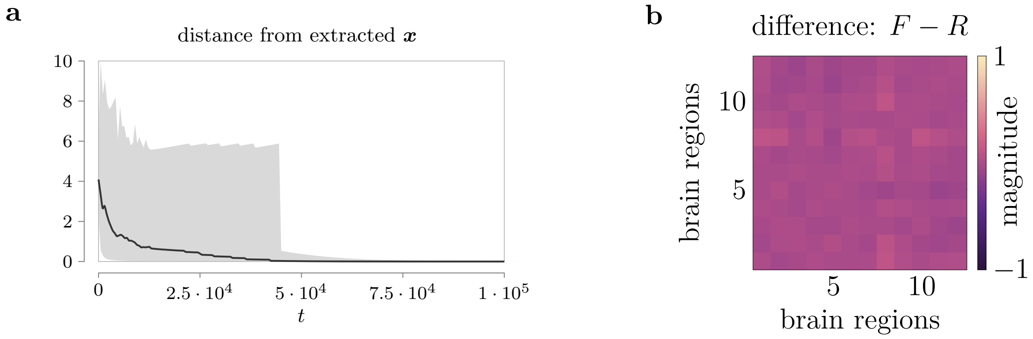

Supplementary Fig 7. Additional analysis on the functional pattern R in the brain network application. **a** Stability of the prescribed functional pattern R from random initial conditions $\boldsymbol{x}_0$ satisfying $\|\boldsymbol{x}_0 - \boldsymbol{x}\|_\infty \leq \frac{\pi}{2}$. The thick black line represents the average ℓ_2-norm distance over 10^4 random initializations, and the shaded area represents the smallest and largest value of the ℓ_2-norm distance. The sudden drop in the largest norm is due to the phases $\boldsymbol{\theta} \in \mathbb{T}^n$ evolving in the torus, thus taking values in the interval $[0\ 2\pi)$. Our numerical simulations reveal that phase trajectories starting from the other half of the torus (i.e., $\|\boldsymbol{x}_0 - \boldsymbol{x}\|_\infty > \frac{\pi}{2}$) may converge to different stable patterns, which are not compatible with the phases extracted from the functional MRI recordings. **b** The difference between the functional connectivity F and the functional pattern R. The entries with the largest magnitude are ≈ 0.08, highlighting the stark similarity between the two correlation patterns R and F.

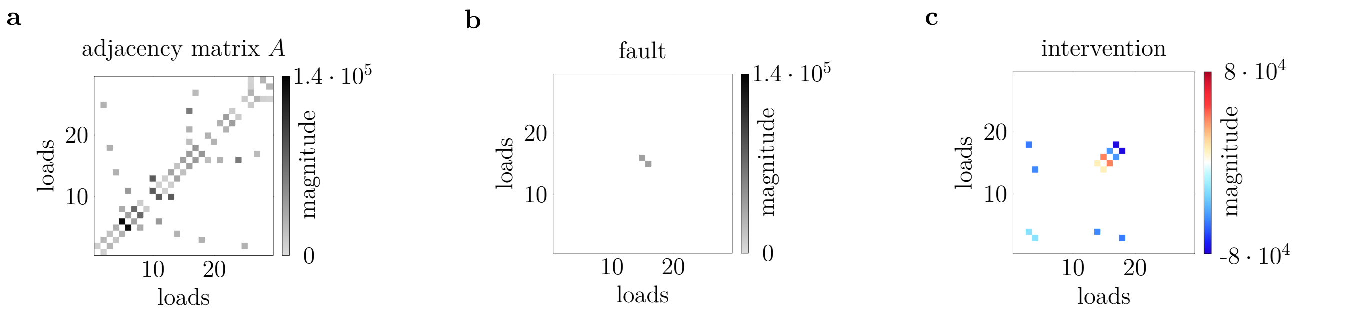

Supplementary Fig 8. Matrices that describe the network interconnections, the fault, and the local intervention of the power network parameters to recover the pre-fault power distribution. a The adjacency matrix used in the Kuramoto model to simulate the IEEE 39 power network. **b** The fault that disconnects loads 13 and 14. **c** The intervention is localized, in the sense that only branches of the loads connected to the ones affected by the fault and their immediate neighbors require adjustments. The sparsity of the local intervention is promoted by the usage of the ℓ_1-norm in the optimization problem.

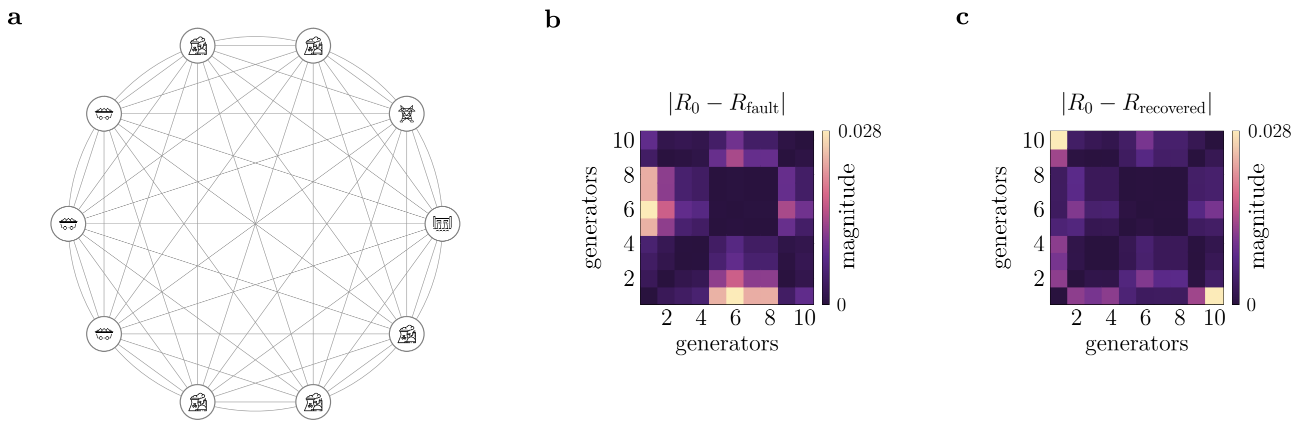

Supplementary Fig 9. Application of our procedure to the third-order model of the IEEE 39 New England. a The IEEE-39 New England power network reduced to a 10-generator network. Electrical loads are simply modeled as passive impedances. In order to explicitly account for the outside of the system, Generator 1 is assumed to be connected to an infinite bus and has constant phase and frequency [28]. **b** The absolute difference between the pre-fault functional pattern R_0 and the post-fault pattern R_{fault}. The Frobenius norm of this difference is $\|R_0 - R_{\text{fault}}\|_{\text{F}} = 0.0907$. **c** The absolute difference between the pre-fault functional pattern R_0 and the recovered pattern $R_{\text{recovered}}$ after tuning the power $\boldsymbol{p}_m$ at the generators. The Frobenius norm of this difference is $\|R_0 - R_{\text{recovered}}\|_{\text{F}} = 0.0653$.

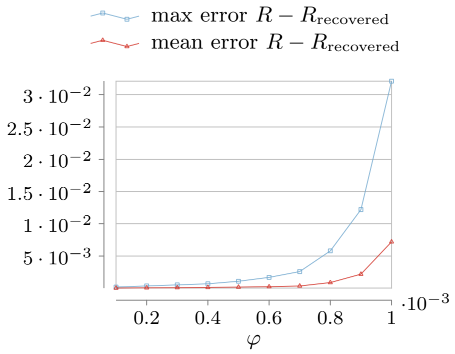

Supplementary Fig 10. Error between the pre-fault functional pattern R and the functional pattern $R_{\text{recovered}}$ obtained through our procedure as a function of the phase shift $\varphi \in \mathbb{S}^1$. The functional pattern $R_{\text{recovered}}$ is computed after a network correction due to a fault that occurs in the IEEE 39 test case between loads 13 and 14 (the same as in the main text). In the presence of a phase shift φ, the error between the desired functional pattern and the one associated with the network correction computed by our method remains small for small values of energy loss φ. Here, the parameter optimization does not explicitly account for the phase shift φ. The mean error is computed as $< \text{vec}(R) - \text{vec}(R_{\text{recovered}}) >$, and the maximum error as $\|\text{vec}(R) - \text{vec}(R_{\text{recovered}})\|_\infty$, where $\text{vec}(\cdot)$ denotes the vectorization.

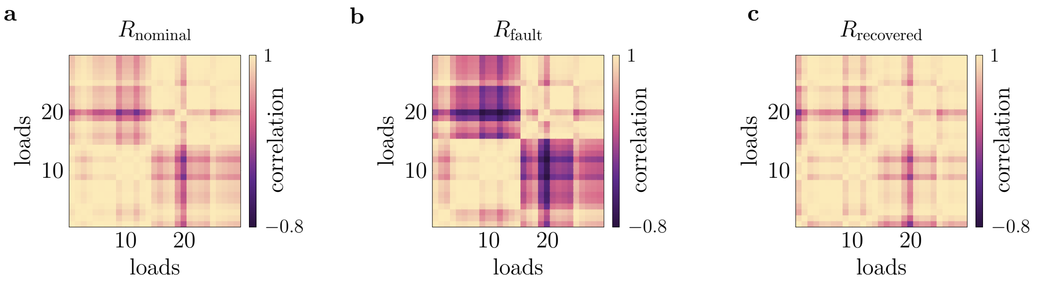

Supplementary Fig 11. Nominal, post-fault, and recovered functional pattern in a network with lossy communications. In all panels, the loss is fixed to $\varphi = 0.01$. **a** Functional pattern associated to nominal power flow in the IEEE 39 New England test case. **b** Functional pattern associated to a power flow disruption due to a fault that disconnects loads 13 and 14. **c** The recovered functional pattern after our procedure with updated constraint (equation (15)) is applied. The mean error between the pre-fault and the recovered functional patterns is $< \text{vec}(R) - \text{vec}(R_{\text{recovered}}) >= 0.072$, where $\text{vec}(\cdot)$ denotes the vectorization of the patterns.
